# State-of-the-Art Sensors for Remote Care of People with Dementia during a Pandemic: A Systematic Review

**DOI:** 10.3390/s21144688

**Published:** 2021-07-08

**Authors:** Chandan Kumar Behera, Joan Condell, Shirin Dora, David S. Gibson, Gerard Leavey

**Affiliations:** 1 Intelligent Systems Research Centre, Faculty of Computing, Engineering and Built Environment, University of Ulster, Northland Road, Londonderry BT48 7JL, UK; behera-c@ulster.ac.uk (C.K.B.); s.dora@ulster.ac.uk (S.D.); g.leavey@ulster.ac.uk (G.L.); 2 Northern Ireland Centre for Stratified Medicine (NICSM), Biomedical Sciences Research Institute, University of Ulster, Altnagelvin Area Hospital, C-TRIC Building, Glenshane Road, Londonderry BT47 6SB, UK; d.gibson@ulster.ac.uk

**Keywords:** sensors, dementia, caregiver, COVID-19, pandemic, assistive technology

## Abstract

In the last decade, there has been a significant increase in the number of people diagnosed with dementia. With diminishing public health and social care resources, there is substantial need for assistive technology-based devices that support independent living. However, existing devices may not fully meet these needs due to fears and uncertainties about their use, educational support, and finances. Further challenges have been created by COVID-19 and the need for improved safety and security. We have performed a systematic review by exploring several databases describing assistive technologies for dementia and identifying relevant publications for this review. We found there is significant need for appropriate user testing of such devices and have highlighted certifying bodies for this purpose. Given the safety measures imposed by the COVID-19 pandemic, this review identifies the benefits and challenges of existing assistive technologies for people living with dementia and their caregivers. It also provides suggestions for future research in these areas.

## 1. Introduction

In this paper, we provide a systematic review of existing assistive technologies that are being designed, created, and evaluated for people living with dementia (PLWD) and their caregivers. In particular, we focus on devices which can be useful in supporting PLWD remotely in the light of physical restrictions imposed by pandemics such as COVID-19. We searched three broad databases online and filtered the articles by relevance to finalize the documentation for this work. We analysed research works published in this area over the last 20 years, and highlight needs for future research.

In the United Kingdom (UK) in 2019, 5% of people over 65 years of age have been diagnosed with dementia and 30% of people over 95 years of age have this disease [1,2]. From 2018 to 2050, the number of PLWD in Europe is projected to grow from 3.3 million to 8.5 million [3]. This would lead to a large rise in healthcare costs, a decline in the standard of treatment, and increased pressure on caregivers because of a shortage of resources [4]. Direct costs are projected to be at least GBP 3.3 billion per annum (circa USD 4.57 billion or EUR 3.85 billion per annum) for the UK National Health Service and social services [5,6]. In addition, the World Alzheimer Report suggests that 115 million people will be affected with dementia by 2050, highlighting the need to improve assistive technology in order to reduce the burden on caregivers [1]. Furthermore, many elderly people tend to stay in their home settings [6] because of the high costs associated with access to care homes and private hospitals, or the lack of adequate facilities. This scenario brings added pressure on the family and caregivers of PLWD. Assistive technology (AT) encourages independence and allows people with physical or cognitive limitations to remain at home [7,8] by maintaining or improving their independence, safety, and wellbeing [9]. In this review, the term assistive technology is used to describe electronic devices that can be used to support PLWD’s lifestyles. These devices can improve the living standards of PLWD, encourage independence, and may decrease hospital admission rates [9,10,11,12]. Furthermore, assistive technology can reduce the stress of caring for PLWD [13,14].

Pandemics such as COVID-19 can cause serious health risks, especially for the PLWD. It has been reported that both the carers and the PLWD experience critical levels of psychological stress during COVID-19 [15,16]. Extra support is needed for the PLWD during such pandemics [17,18,19,20,21,22,23]. Although the usage of assistive technology (AT) can help reduce the burdens of carers and reduce the health risks of the PLWD, it may not fully address the needs of caregivers and PLWD during a pandemic when there is a need for minimising physical contact [15,22]. For instance, studies indicate that COVID-19 lockdown constraints can trigger depression, loneliness, and anxiety as a result of limited social activities and family interactions [19,20,21], and this may impact more on vulnerable populations such as PLWD [22,24,25]. AT devices that support remote assistance of PLWD [26,27] can play a vital role in mitigating loneliness and stress caused by pandemics, reducing the need for home visits and hospitalisation, thus reducing the costs associated with caregiver services. Reducing the risks of virus transmission within care homes are also a major consideration [28]. AT can assist in maintaining PLWD at home and preventing admission to hospitals [29]. However, there appears to be a lack of awareness about the availability and utility of such devices among PLWD and carers [30,31,32], and the stigma associated with using certain AT devices may also discourage use [33,34].

There is a need for further research and evaluation of AT in dementia care across diverse settings. For instance, devices that use cloud computing are not reliable where there is a lack of fast and stable internet access. As a result, remote monitoring devices requiring cloud computing need to have an alternative mechanism to perform their functions when the connectivity strength is low. Furthermore, certain remote monitoring devices need a computer access at the locations of both carers and PLWD [27], which may limit use. This review describes the potential benefits and pitfalls of the assistive technologies available for PLWD in the context of a pandemic and provides future directions of research for this area of study. While this review is not a checklist of all the assistive technologies for PLWD, it will provide some knowledge on current technical solutions based on diverse needs. To the best of our knowledge, this is the only review work which provides currently existing technical solutions as per the needs of the PLWD and their carers during a pandemic situation, and for the physical restrictions that have been imposed during COVID-19.

We have found several studies evaluating the effectiveness of AT devices for PLWD and carers [31,32,35]. For example, one recent study assessed the impact of AT on the safety of PLWD who are not under institutional care [34]. The authors highlighted that the available evidence at the time of their study did not conclusively indicate that AT improved the safety of PLWD living outside care homes. They built upon a previous work by updating the safety aspects, and highlighted similar conclusions [36]. The authors mentioned in their review that the existing AT devices for PLWD lacked clinical trials with required sample sizes. Compared to their studies, our review has an even broader focus as it involves ATs and services that aim to improve the independence, safety, and/or quality of life for PLWD. For instance, the above studies did not introduce and describe various AT devices available for PLWD and carers. Nor did they describe the future works on the AT devices that can have significant improvement in the usages of AT devices by the PLWD and carers. We have summarised such aspects in Table 1 of this review. Similarly, there are studies which did not include any standard research trials evaluating the effectiveness of AT devices for PLWD [26,27]. It can be noted that these studies lacked randomized control groups and use of quality indicators, and had small sample sizes, e.g., one study advocated the usage of AT devices by PLWD and carers based solely on semi-structured interviews of 13 PLWD [32]. Similarly, another study identified only three clinical studies involving PLWD (i.e., COACH, CareWatch, and CareMedia), but with small sample sizes (e.g., clinical trials on COACH were conducted on 10 subjects, and CareMedia was conducted on 15 subjects) [35]. In a recent review work, the authors provided a broad spectrum of available AT devices for the PLWD during the time of their review. While providing details about AT devices for PLWD, only 1.1% used a randomized control trial design, and 51% had no testing done on PLWD. Moreover, these trials had small sample sizes of typically less than 20 people [37]. Among the ATs which received clinical validations, most of them (n = 254) had small sample sizes (n < 20). The authors showed that during the time of their systematic review, they had found that more than half of the ATs did not receive clinical validations through human subjects. Thus, we noted that, despite being marketed or developed for PLWD, a range of AT devices are produced without a validation that they work for PLWD. Such factors restricted our confidence in the outcomes of some studies. We report on these studies, but we have also mentioned about our confidence in their outcomes based on their quality indicators.

In our study, we have completed a focused review work, where we targeted only a specific type of ATs and services, which have remote facilities for the PLWD and carers. In our review work, we included many recent Ats and services, and their studies from relevant recent publications. Some of the publications which we have referred in our review work were published based on the recent pandemic and were relevant based on the context of our study. Another new study, although covering a broad range of AT devices for the users, focused mainly on the experience of informal carers to support PLWD living at home using AT [38]. Furthermore, they did not include the quantitative studies, rather providing a narrative review. Moreover, the requirement of awareness of technology among the users and the possible solutions are not described in detail.

Our review work has bridged upon the above gaps of the previous studies. Furthermore, there have been previous pandemics, spread mainly because of person-to-person physical contact [39]. Hence, we have gathered evidence on AT devices with remote access facilities that can be helpful for the users during the physical restrictions imposed by a pandemic scenario, e.g., COVID-19, as there have been no review works conducted to take into account the above important aspects.

The next part of this systematic review describes the materials and techniques, which we used for conducting the review of published articles based on currently existing AT devices meant for PLWD, which are to be assessed remotely by the carers or the clinicians. Then, we report the results of our review, describing the available technologies falling in this domain. Finally, in the discussion section, we summarize the results of the review and provide future research priorities.

## 2. Materials and Methods

**Figure 1:**
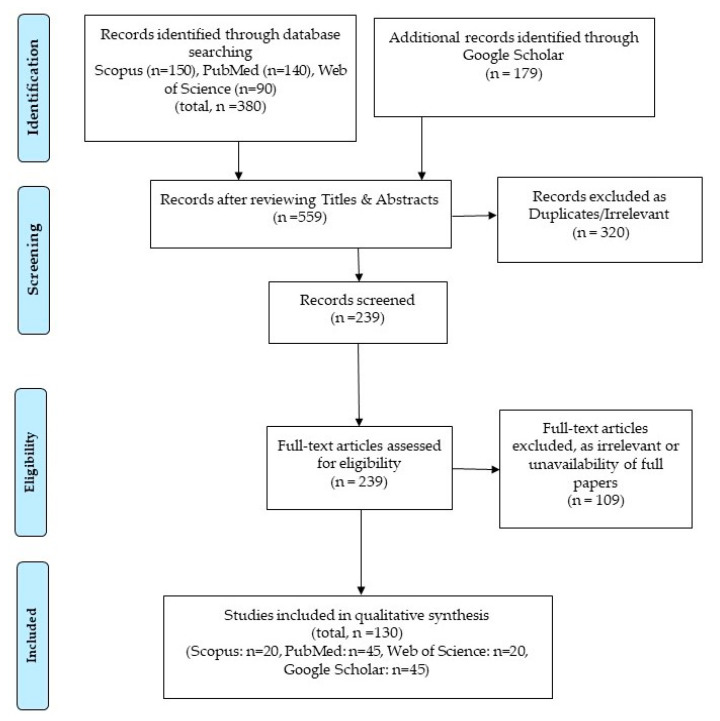
Process for selecting studies included in this review using PRISMA guidelines [40].

This review focuses on the types of technology that are designed, developed, and evaluated, keeping in mind the abilities of carers to remotely monitor daily activities of PLWD that would provide them an independent life. We followed the Preferred Reporting Items for Systematic Reviews and Meta-Analysis (PRISMA) guidelines for conducting this review (refer Figure 1). The PRISMA checklist is included as a supplementary file with this review. This review is based on a systematic search for relevant articles published in the last 20 years in three databases, namely Scopus, Web of Science, and PubMed.

### 2.1. Search Strategy

We used Google Scholar for our search, as well using its “advanced search” option. Only the articles published within the past 20 years were included. The phrases/keywords searched are: (“Assistive Technology” OR “Assistive Device” OR “Adaptive Technology” OR “Adaptive Device” OR “Enabling Technology”) AND (“Dementia” OR “COVID-19 restrictions” OR “Aged” OR “Home” OR “Elderly” OR “Caregiver”), while searching for the required articles. In PubMed, the medical subject headings (MeSH terms) used were “Dementia”, “Caregiver”, and “Home”, whereas the non-MeSH terms were “Aged”, “Elderly”, “AT device”, “Adaptive Technology”, or “Adaptive Device”. We looked for devices or services that can be specifically used to control or monitor activities of PLWD remotely to provide them independent living, reducing the burdens of the carers. While searching for the articles we paid special attention to the connecting phrases between the words, because different combinations of the phrases can produce different search results. For example, if “or” was not used specifically, then the repositories used “and” by default to join them, and the searching process required modifications. Furthermore, quotation marks were also used in order to find the exact phrases in the articles. No hyphens, commas, or apostrophes were used, as they would produce the same search results without any improvements. The above synonyms were automatically identified by PubMed in the section labelled as “Entry terms”, and they were repeated for searches in the other databases, such as Scopus. The results were sorted by newest, most cited, and relevance. All the searches were conducted in the English language only. In this search strategy, a total of 559 articles (150 from Scopus, 140 from PubMed, 90 from Web of Science, and 179 through Google Scholar) were identified for consideration.

### 2.2. Selection of Articles

Prior to reviewing the papers, a two-stage screening process was followed to identify the articles relevant for this review. During the first stage of the screening process, the titles and abstracts of the papers were reviewed. A total of 320 articles were considered irrelevant or duplicates (104 from Scopus, 67 from PubMed, 63 from Web of Science, and 119 from Google Scholar). The relevancy of articles at this stage was decided based on the central theme of our review work, which was to identify articles describing devices/services for the PLWD and carers which would be helpful during a pandemic scenario enforcing social distancing and other safety measures based on physical distance. In the next stage of the screening process, the full texts were studied along with the abstracts. If the full texts were not available online, authors were contacted. In the case of no replies, they were not included in the review. Some papers on assistive technologies were not specifically meant for dementia or remote sensing devices. Those articles were not relevant for our review. A total of 109 articles were rejected as irrelevant at this stage (26 from Scopus, 28 from PubMed, 7 from Web of Science, and 15 from Google Scholar). Finally, 130 articles were selected for review based on their relevance to the topic of this paper. The papers were searched by the first author. Two researchers (first and third authors, C.K.B. and S.D.) independently conducted the screening with the exclusion and inclusion criteria. The records in disagreement during the abstract screening and selection of full texts were discussed between the two researchers. Since the two researchers resolved all the conflicts about the inclusion of full texts for the review, a third reviewer was not required to be involved to judge the relevancy of articles for the review work. All the co-authors agreed for the contextual appropriateness of the selected articles. The manuscript was edited by all the co-authors during several instances of the work.

### 2.3. Synthesis of Data

The devices/services described in the articles as identified after the screening process were divided into three different categories based on their usage scenarios. The articles that did not fit in any of these categories were assigned to a category, namely “Miscellaneous”. If an article could be assigned to more than one category, then it was associated with multiple categories. Table 1 shows the categories for each of the articles selected after the screening stage, along with a brief description of the functionality presented in that article.

**Table 1:** Details about the categories and functionality of each article reviewed in this paper.

Device/Service	Category	Functionality
Cognitive Assistive Technologies (COACH, zero-effort technologies) [41,42,43]	Telecare	Prompt to PLWD on daily activities at home (such as hand washing).
Smart home [44,45,46,47,48,49,50,51,52,53,54]	Telecare	Analyse health, prompt for medication, support for cooking, dressing, clinical trials at home.
Avoiding hospitalization [55,56,57,58]	Telecare	Home care, (cost-effective, health safety, psychological stress).
Virtual visiting (Social robots) [9,19,36,51,59,60,61,62,63,64,65,66,67]	Telecare	Remote health check-up, reducing physical contact, cost-effective informal caregiving (supporting food preparation, eating, recreational activities).
Reminder systems [53,54,68,69]	Telecare	Memory support (alarm, communicator, diary, reminders), remote schedule prompter, medication regime support.
Video monitoring system [19,26,38,70,71,72,73,74]	Telecare	Audio-video conferencing with family and remote surveillance.
GPS-based devices [75,76]	Location	Monitoring geographic location of PLWD.
Safe walking [77,78,79,80]	Location	Navigation and route finding.
Smart phones with GPS [80,81]	Location	Navigation prompting with identification of indicators of disorientation for PLWD.
Exercising in virtual environments [82,83]	Location	Indoor exercising, navigating practice.
Health monitors [84,85]	Location	Health monitoring in outdoors activities.
Safety keys [86,87]	Safety and Security	Easy access to house.
Auto water controller [86,87]	Safety and Security	Automatically disables water flows.
Auto gas supply controller [86,87]	Safety and Security	Automatically disables gas supply.
Safe home (door alerts, video call, burglar alarm, automatic fire detectors) [88]	Safety and Security	Remote home surveillance.
Social alarm system (crime surveillance, fire alarm services, community safety, telehealth) [89,90]	Safety and Security	Social support to PLWD during emergency.
Pressure mats [91,92]	Safety and Security	Movement detection and automatic communication to the carer.
Telephone-blocker [86,93]	Safety and Security	Blocks unwanted calls automatically.
Item locator [10,24,94,95,96]	Safety & Security	Helps locate important items for PLWD.

## 3. Results

Several technological solutions exist for PLWD and their carers [97,98,99]. Some of these solutions have no predefined interaction or expect any pre-existing knowledge from the PLWD. Such solutions may not only be helpful from a safety point of view, but also could help to reduce the emotional stress of carers [31]. For instance, technologies with video conferencing (such as Skype, FaceTime, etc.) help PLWD socialize with family and friends, thereby imparting positive emotions and reducing anxiety and agitations [72,73]. Furthermore, one of the recent review works has presented the existing technical solutions to aid communication skills for the people with dementia [100]. The review work pointed out that the PLWD lack communication skills, which hinder their social interactions. Although there are a few technological interventions in this regard, the use of technical devices to aid communication skills are still in their infancies, e.g., tablet computers can be used as a digital photography diary by providing snapshots of the day while viewing them along with the friends and families. This can increase social interactions even if the PLWD lacks communication skills. Similarly, social robots such as PARO, and computer games such as MARIO, can allow flexible interactions. The studies included in this review, however, lacked control group and had small sample sizes. More research is needed in this area to promote social interactions among the PLWD. For the PLWD who suffer from language impairments, certain technologies such as electrooculography (EOG), eye-tracking, and electroencephalography (EEG) can be used to capture the body movements and send the commands to the computer, facilitating human–computer interactions [101,102]. However, EOG- and EEG-based devices have a requirement of mounting electrodes on the subject’s head, which can be bulky and uncomfortable, particularly for the PLWD. Furthermore, the camera-based devices can cause privacy concerns. Hence, systematic trials of such devices are needed at this stage. On the other hand, there are certain technological interventions such as radar-based biomedical applications, which allow human–computer interactions [103]. The application can simultaneously interpret the head movement and eye blinking of the subject, which helps him/her interact with the computer. This can aid the PLWD who suffer from language impairments. However, the research in this area is at a very preliminary stage. More technical improvement, followed by user testing, is needed. Cognitive assistive technologies have emerged to address prompting of everyday tasks for PLWD [41]. For instance, in order to assist the PLWD with hand washing, there are technologies such as Cognitive Orthosis for Assisting Activities at Home (COACH), which can provide audio–video prompts for the process [41]. COACH is regarded as a “zero-effort technology” because PLWD need to provide almost no effort to use such technologies [43]. By using artificial intelligence and computer vision, COACH detects the activities of PLWD and provides prompts during various stages. Recent research focuses on incorporating personality and emotional models into such prompting structures to easily incorporate them into the lives of PLWD [104]. “Smart homes” use such cognitive assistive technologies with the help of artificial intelligence and several sensors to analyse the health, and detect the activities, of PLWD at home [45]. They can automatically monitor behaviour and health, and send the information remotely to carers, clinicians, and social organizations [46]. For example, the Gloucester Smart Home provides automatic prompts, both visually and verbally to the PLWD, of certain messages such as “it’s time for your medication” [47]. Recent sensor technologies have adopted advanced artificial intelligence techniques to provide prompts and supports in several day-to-day activities of the PLWD, such as cooking [48] and dressing [49], and they help in reducing the burdens on the caregivers [46]. They do not require personal help to install the devices at home [50]. Furthermore, these smart homes even provide a platform for conducting clinical trials within the home settings [52,53]. In a pandemic scenario, such technologies can potentially help reduce hospitalization by reducing the risks of getting infected and spreading it to others. Moreover, the carers can particularly benefit from such technologies, because they can remotely monitor the activities of PLWD, which is an important aspect given the need to minimize physical contact during a pandemic, e.g., COVID-19.

Developments in computing technology have increased the usability of the devices meant for PLWD and have improved the effectiveness of devices for remote monitoring of the activities of PLWD by carers and clinicians. Cloud computing in dementia research allows faster computing along with reduced cost to healthcare providers, which is important in health-related emergencies [105]. Such technologies are used in a variety of dementia supports, such as activity monitoring, location tracking, analysis of neuroimaging data, etc. [74,76,106]. Table 1 presents the details of the technologies offering remote functionalities for PLWD and their carers.

These assistive technologies can be useful for PLWD, given the physical constraints imposed by a pandemic such as COVID-19. These technologies or devices alleviate the need for physical contact with the patients and can be broadly divided into three categories, as per Table 1 [86]:

- Telecare.
- Location.
- Safety and Security.

### 3.1. Telecare

Telecare devices can be installed in homes to enable remote execution of a variety of tasks by application of several technologies. Telecare services such as “electronic assistive technologies” and “environmental controls” are used to render a full framework of “smart housing” [44,46]. Studies indicate that telecare technologies that require minimal interaction from PLWD are more successful, because of their impact upon quality of life [44]. These devices also facilitate remote surveillance of PLWD in their own homes and provide them with greater independence in their daily activities [86]. Most of these telecare devices use several sensors, including alarm systems, to provide real-time information to the caregivers remotely. These telecare systems also include fall detectors. Such telecare services provide valued independence. In the case of dementia, however, proof of cost-effectiveness is lacking, as current research lacks adequate user, cost–benefit, and health economic testing with PLWD in particular [101].

Another important category of telecare devices are those that enable clinical processes to be conducted remotely [44]. Such devices use advanced technologies for data processing (such as voice recognition and visual image processing). Other factors, such as artificial intelligence (AI), mobile systems, and remote sensors to diagnose and monitor the treatment of patients, have improved the utility of telecare devices. Hence, telecare systems can enhance services available to PLWD and take into consideration any restrictions on physical distance, as they operate remotely. Below, a description of different telecare devices has been provided, categorized according to their functionalities.

#### 3.1.1. Reducing Hospitalization

Telecare services can help to significantly reduce length and number of hospital admissions. The average hospital duration of stay could be shortened by between 20% to 60% [54]. Telecare services can also support the independence of PLWD by providing some hospital facilities at home. Hospital care provides around-the-clock care during a period of hospitalisation; however, assistance can be limited by available resources soon after discharge. Telecare services can also be considered as a form of interim treatment, such that once discharged from hospital, PLWD can continue to be provided with a level of treatment or monitoring using home technology.

Such services may reduce costs associated with treatment by providing economic treatment alternatives applicable to the home setting of PLWD and reducing transfers and handovers involved in patient admissions. This approach could greatly help to work within the social distancing constraints imposed by a pandemic such as COVID-19. In addition, it might also reduce the tremendous pressure on healthcare services during a pandemic [55]. This could be particularly beneficial for PLWD in whom poor outcomes result from acute hospital admissions due to increased risk of behavioural and psychological disturbances during such stays [55,56]. Studies suggest that acute admission to general hospitals should be avoided to reduce associated risks of delirium and to maintain a stable mental state [55]. Therefore, based on the pandemic scenario created by COVID-19, avoiding hospitalization is desirable especially for PLWD, who may also be at increased infection risk due to reduced protective immune response evident in older PLWD [58].

#### 3.1.2. Virtual Visiting

Virtual home visits of PLWD dramatically reduce mortality (by approximately 25%) and long-term care admissions (by approximately 45%) [7]. Such assistance, however, has been withdrawn in Wales and England, because of the considerable personnel requirements associated with them. Therefore, due to cost and personnel constraints, home visits are not undertaken as much as desired. As an alternative approach, telecare devices offer an effective solution, which is particularly helpful to PLWD living alone. This approach can go some way to address user and professional satisfaction [56]. Studies from a community nursing research perspective have shown that up to 46% of home visits can be replaced with virtual visiting [7]. Furthermore, it can help reduce the need for physical contact in a pandemic situation imposed by a disease such as COVID-19.

Robots have also been developed to support the caregivers [46,52]. Social robots can remotely monitor the activities of PLWD using several sensor types and provide prompts through video conferencing [60,61,64]. These robots can support PLWD in daily activities such as food preparation, eating etc., along with providing opportunities for recreational activities and informal caregiving [42,59,65,67].

#### 3.1.3. Reminder Systems

Telecare systems can serve several purposes, including as a reminder support system. Short-term memory loss, for example, is commonly observed as part of the normal aging process, but it can easily curtail independence for PLWD. The functionality of an alarm, communication system, diary, and platform for setting reminders can be combined with basic technologies [69]. When such technology is combined with the capabilities of mobile phones it can help caregivers easily keep in touch with PLWD. A recent research example suggests a remote reminiscence conversation and prompter system which works via a videophone device [68].

Reminder systems help people manage their medications, as lack of compliance with medication regimes is a common issue amongst PLWD. Automated pillboxes can be operated from a small workstation at the PLWD’s home and provide prompts from remote call centres or carers as to when medication is due [46,54]. In a recent report, it was shown that such systems can decrease hospitalisation rates by 41% and improve drug enforcement from 34% to 94% [46,54]. Due to healthcare and social constraints imposed during the spread of COVID-19, caregivers can benefit from the applications, such as reminder systems, through remote implementations.

#### 3.1.4. Video Monitoring

Information and communication technologies can provide real quality of life improvements for PLWD [26,70]. Video monitoring can serve the need of improving safety in real time by exploiting advances in communication technologies, networks, and developments in video and audio processing algorithms over normal telephone lines [69]. Although the primary use of such technology, commonly via touch tone telephone, is remote surveillance, it can also be used for video and audio communications with family members. As there is a need for computers or mobile devices at either end, usability can be difficult for PLWD. Video monitoring systems can be linked to social caregivers to assist with remote services during periods of restricted care. For instance, these systems can be used to trigger emergency services to respond to a fire alarm and inform the caregivers in real time, providing them with an opportunity to remotely monitor the situation and provide support. Given the current scenarios created by the COVID-19 pandemic, such video monitoring systems could also be of great help in remotely monitoring the daily activities of PLWD, along with maintaining the physical constraints imposed by it.

### 3.2. Location

Location monitoring normally uses Global Positioning System (GPS) technology to remotely check the physical locale of PLWD by caregivers [75]. Although many studies show that caregivers and PLWD alike find these systems helpful in providing a level of independence [78], there are some contradictory studies which show that with daily usage, PLWD have less confidence in them [102].

Outdoor activities are important for the health benefits of PLWD. Various technologies such as smartphones with a GPS tracking facility and maps support “safe walking” and help with navigation for PLWD, which is often challenging when they are outdoors [77,78,79,80]. Furthermore, there are virtual systems which provide such facilities for the indoor environment, e.g., exercising and navigating virtual home settings [82,83]. PLWD who have mobility issues can use virtual environments as an alternative to carer-supported exercises, which is potentially beneficial to both the carers and PLWD. This is particularly beneficial during pandemics such as COVID-19, which require physical restrictions as safety measures.

From our literature review, we found no randomised control trials which investigated the efficacies of location-based technologies for PLWD. However, there are a few trials with positive outcomes. A recent trial was conducted over three months, with 28 PLWD and their carers. In this study, 77% of carers recommended to others the benefits of GPS technology [107]. Around half of the PLWD found that having a GPS system indicated that they became more unaccompanied, whereas 45% reported that they were more often left free, and 25% reported to be more regularly left free. Furthermore, half of the carers reported a positive impact with reduced stress levels after the trial. Moreover, it was reported that the carers could provide more freedom to their loved ones (60%). Hence, it has been shown that the GPS technology has several beneficial effects on the PLWD and the carers using it. In their study, there were two dropouts, because of their dementia progression. The PLWD could not go out alone, and therefore there was no need of the GPS technology to track their locations. This was the same reason for the dropouts in a different such trial testing the efficacy of GPS technology on PLWD [108]. This shows the downside of GPS technology, that it cannot always reduce the risks among the PLWD to go outdoors alone and, hence, while introducing such technologies, more than just the potential of the person to find his or her way back home should be considered. For instance, GPS technology cannot minimise the risk of road accidents, in case the PLWD is vulnerable to such risks.

In other studies, it is shown that the GPS technology might also offer a false sense of protection. In other words, it provides an impression that the individual using it is inherently “safe”, while several other risk factors still remain at large [108]. Therefore, it is mentioned that early stages of dementia are the suitable periods during which such technology can be helpful for the PLWD; however, we believe that it can also be used in later stages of dementia to reduce the risk of wandering, because of its various functionalities. For instance, it can be used to improve safety for the PLWD to encourage the freedom of roaming in all stages of dementia [108,109]. In this study, some practical issues which should be noted while using GPS technology have also been presented. Many users did not actually carry the GPS device while going outdoors (33% of the time), for several reasons such as being familiar with the everyday routes and low battery of the device. Moreover, in this study, forgetfulness in carrying the device before going outdoors was not reported to be an issue, although it could be an issue for the PLWD living alone. It is shown in other studies that efficient usage of modern AT devices relies on the consistent usage in the everyday routines of the PLWD [110,111]. As a result, making it a habit to charge the device and carry it while going outdoors is crucial in accessing the advantages of GPS technology in fulfilling the needs of the PLWD and carers. Another trial was conducted to test the efficacy of location-based technology [112]. The study was conducted over a period of 3 years, consisting of 25 Finnish PLWD with their carers, and it was shown that ATs such as location-based devices and motion sensors were most helpful for the PLWD to stay home. This is because, during the study period, such technologies could help avoid potentially harmful events, for instance night wandering during winter. Because of the frequent changes of the requirements of the users, various types of the AT devices were used in the trials for an average period of 7.5 months. This study shows the frequent changes of the technological requirements of the PLWD. For instance, ATs such as GPS devices and motion sensors can be helpful only for a particular period of time, when the PLWD is still able to be mobile and maintain his/her safety while being outdoors unaccompanied.

In another study, conducted over a period of 2 years to test the efficacy of GPS technology among the PLWD, several important themes were reported based on qualitative interviews [113]. In this study, it is reported that the carers found the GPS technology to be very much advantageous in terms of improving not only the safety but also the freedom. Furthermore, in a different study concerning GPS technology and dementia, it was shown that in order to ensure the safety, the carers would have to consider restricting the mobilities for their loved ones had there been no such technology [108]. This freedom was highly valued by PLWD. In addition, it is reported in the study that the PLWD who used such technology did not have the feelings of being monitored or tracked. This might be because of the particular usages of the carers, because the carers reported using it only when necessary, instead of infringing on the privacies of the persons. This shows that for the device to be successful and beneficial for the users, the privacy of the PLWD plays an important deciding factor. Furthermore, it was reported that even if the participants faced some technical challenges, they found the GPS technology to be very much helpful. Another study was conducted on five pairs of spouses with mild to moderate stages of dementia, for a period of six weeks using GPS technology [109]. The participants were not only interviewed, but also observed during the study. It was reported that all the participants found the GPS devices to be very much helpful, particularly in supporting the freedom of PLWD. The participants had developed a good level of reliability and an increase in their confidence in using the GPS devices through repeated testing. This is an important aspect for efficient use of the devices, i.e., to be familiar with the usage of technology through practice. In general, the study reported positive feedback about the usages of the GPS technology. Even if some participants needed the support from the researchers towards understanding the technology, all of them could finally adopt the technology. Apparently, the study showed that the key role of GPS technology was for using it for safety of the PLWD while being outdoors, instead of using it as a precautionary measure for wandering. In this study, it was shown that the PLWD did not have privacy infringement issues of being monitored, whereas there are other studies have reported such issues [113,114]. This is also emphasized by professionals in the literature [115,116]. However, we think such concerns about privacy infringement may not be a deciding factor for the usage of AT devices, as the PLWD may not always have the capacity to consent, given the benefits of AT devices towards safety and security of the users. In fact, it is reported that the participants wanted to be locatable and were becoming concerned when they were not seen [117]. This might be because in the study, the location information was shared only with the spouses.

Finally, another study was conducted on GPS technology, with 18 PLWD and carers, over a period of 2 months, showing the comparison between the usages of two different GPS watches. In that study, both the types of GPS watches were tested by the same participants; hence, they could report the comparative usability of both devices. The usability of the two devices varied based on the ratings of features of the devices. Hence, the crucial message from the research on GPS devices is that the efficacy and usability of the existing GPS devices differ from manufacturer to manufacturer [118].

To summarize, the findings from these studies were limited by the absence of a control group, and small sample sizes. Additionally, the indicators were of low quality and minor statistical significance. Hence, we consider the results of these studies to be only partial in examining the efficacies of location-based technology for PLWD. However, GPS technology may be very much helpful for the users and carers in maintaining dementia-related behaviours such as agitation and wandering.

#### Health Monitors

Used similar to wrist watches, these devices help monitor the movement, skin temperature, and pulses of the bearer. With continuous usage, a pattern is automatically generated for the individuals, and whenever there is any deviation it transmits an alarm to the carers through multi-link [84]. Such devices can be used by PLWD to remotely monitor their general wellbeing. They can be further used as fall detectors (e.g., Tunstall, Tele-alarm) and to transmit real-time location information to remote carers [85].

These devices use an accelerometer and a tiltmeter. Importantly, a tiltmeter can determine the orientation of the wearer. When an impact is greater than a predetermined threshold, the accelerometer detects it [85]. These sensors can therefore reduce the possibility of any false alarms.

Currently, three types of such devices are available:

- Tunstall: They primarily detect the impact and then the angle of tilt of the wearer. It generates an alarm after producing a 15 s warning, if horizontal. The alarm can be cancelled during the warning time. Some users find difficulty in wearing it all the time, for example, during bedtimes.
- Tele-alarm: These sensors consist of an accelerometer and a tiltmeter and can measure tilt continuously. Whenever the change in tilt is greater than 45 degrees and is followed by an impact, it generates an alarm. Unlike Tunstall, they can be worn at any time; however, they do not provide any warning of an impending fall.
- Technology in healthcare: It detects the rate of change of body tilt angle. The body tilt angle during a fall provides a measure for the change of posture. When the tilt rate is greater than 30 degrees/second, it generates an alarm.

All the above devices generate alarms when installed in the PLWD’s home, as well as in the remote care centre, through a telephone network. The operating principles of Tunstall and Tele-alarm devices are the same, but their triggering mechanisms differ subtly. These devices can also be used as wandering detection systems, transmitting a radio signal via a multi-link to the community centre whenever a user goes out of the detectable range. Some PLWD do not like the idea of wearing an extra device. Hence, most of these devices come with a button to send the radio signal to a community service. The user still needs to remember to carry it while going outside. In addition, the patients suffering from other dexterity issues or disabilities, such as arthritis, can face difficulties in fitting the device. Moreover, PLWD with hearing problems may not recognise the beep sound to cancel the alarm. Therefore, more research is required to improve the utility of this technology. Since such devices can send the activity information automatically to remote devices, which can be eventually monitored by the carers or clinicians, these are potentially one of the more important devices which could support PLWD and carers during the pandemic situations.

### 3.3. Safety and Security

Such devices are used to monitor the activities of PLWD, which could be sometimes dangerous. For example, water flow or gas supply technology can be used to automatically disable them during emergency situations, security keys can be used for emergency access to the home, geofencing can reduce the risk of uncontrolled wandering, and telephone blockers can be used to redirect or reject unwanted calls that are not in a predefined list [88]. Safety and security are major areas of concern for PLWD, and there are several technologies available to fulfil these needs [87].

Various types of sensors exist for safety and security, which can be placed in several places of the house, such as the under the chairs, beds as pressure mats, or in the doorways and exits [119]. There can be several ways to alert the users when the sensors are activated, e.g., the alert signal can be sent to a different device, such as a buzzer. Such sensor technology, designed with safety in mind, can alert the carers and PLWD in various instances, such as when then the PLWD leaves the bed or opens the door. However, the literature on these devices is scant. Users usually provide positive feedback to such alert systems [9,88,120,121]. Some users also have positive impressions of the devices, such as door alarm and pressure mats, based on their usages [119,121]. Furthermore, participants in one trial found several ATs, movement sensors, and door alarms to be efficient aids in avoiding admissions to the hospital or care homes [112].

#### 3.3.1. Home Security

Several technologies can be used to improve the home security [88]. For example, the door alerts use electromechanical sensors to detect wandering or intrusions and can send an alarm remotely to the local authorities or community centres via a video monitoring system. The infrared movement detectors are also used for the security of PLWD, by sending alarms remotely to the caregivers, and they can be treated as burglar alarm systems. The automated fire alarm can send an alert signal to the fire services remotely in the presence of smoke or an unexpected temperature increase. These systems are also frequently linked to ambulance, rescue, and police services [7]. In a pandemic scenario, the restriction of commercial and community services has resulted in more PLWD working or staying at home, which has amplified the importance of such home surveillances devices for controlling crime and ensuring safety.

#### 3.3.2. Social Alarm Systems

These systems provide necessary help during emergency [89,90]. Rather than being proactive and preventive, such technologies are reactive and sensitive and can be helpful during a time of need. Some studies show that by using such a system, the total number of hospitalisations decreased by 25% and the hospital inpatient days dropped, on average, from 9.2 to 5.7 days [46,54]. The social alarm systems are normally linked with several emergency services, such as fire alarm service, crime surveillance, ambulance service, community centre service, etc. Using the above system, the West Lothian Council has a long-established UK-based project which has demonstrated several benefits [44]. Such systems can also be beneficial in providing services offered by community call centres to PLWD suffering from loneliness and depression. These systems can be especially beneficial in times of a pandemic when social interactions are difficult.

#### 3.3.3. Pressure Mats

The mobility from a chair or a bed of PLWD can be detected by a change of pressure on these mats, through electromechanical sensors. They can send the real-time information to carers through an audio–visual alarm system [91,92,122]. They can be used as both a standalone aid and a communication link with the nurse or carer. They can also serve the purpose of detecting wandering and can be used in fall prevention. Products such as the door alarms and pressure mats were found to be helpful in some particular cases [119,121].

#### 3.3.4. Telephone Blockers

Along with the requirement of precautions related to physical safety, the PLWD are often susceptible to financial risks. One of the ways that criminals (“scammers”) victimize the user is through phone calls. Commonly, details are listed and shared with other scammers. It is reported by around 70% of carers that the PLWD are routinely called by scammers [93].

AT devices have been designed to automatically block unrecognised phone numbers, permitting access only to a “safe list” indicated by the user [86].

#### 3.3.5. Item Locator

Cognitive impairment creates additional anxieties. Around 62% of carers report the loss or displacement of objects by PLWD to be stressful [30]. An item locator can help the PLWD by guiding him or her in locating the desired items. The way an item locator helps the PLWD is through its parts, called a “tag” and a “hub”. The “tag” is a smaller device which the user needs to attach to the item which he/she frequently forgets to locate, e.g., a key or a TV remote. The “hub” is a relatively larger device which is used to communicate with the “tag”. When the user presses a button on the “hub” it communicates with the “tag”, which produces a beep sound to easily locate the lost item. The functionality of item locators is more or less similar in various brands.

Trials on item locators were conducted in the ENABLE project [9,94] by its Irish, English, Norwegian, and Finnish arms; however, the sample sizes were small, so the trials were conducted on a few devices only. The dropouts in the study were because of the high technical faults, leading to frustrations among the participants towards using the item locator. However, as the product was just a prototype produced in 2003, these technical faults might be most likely because of the specific device. The caregivers believed that it would provide independence to the PLWD by showing them the way to locate the lost items, before the trial was conducted in the focus groups [11].

Moreover, it was reported that in actual practice, PLWD did not find the item locator to be easy to use, but rather this required prompts and additional help [11,94]. Some people did report a degree of usefulness [11,96], such as the reduction in search time and stress. In this study, there was no such technical difficulties with the device reported during the trial. Most of the participants used items such as keys and handbags to tag. One caregiver reported that the privacy of the loved ones was preserved because of the item locator, as their belongings did not have to be looked through while searching for the lost item.

## 4. Discussion

Pandemics, and the measures taken to contain them, can cause serious health risks for people living with dementia and their caregivers. It is reported that nearly half (46%) of PLWD suffered from negative impacts on their mental health because of the pandemic caused by COVID-19 [123]. COVID-19 has indirectly affected the ability of PLWD to access normal clinical services, and the carers are also exposed to critical levels of psychological stress, as the external support structures are more difficult to maintain with added primary healthcare burden [14,107]. Several recent studies indicate the importance of sufficient care for PLWD during COVID-19 [16,17,18,23,124]. A recent study shows how technology can be used to deliver a group of interventions to PLWD during the COVID-19 pandemic [20]. Another recent report showed that because of the COVID-19 pandemic, there is an increase in symptoms suffered by the PLWD, e.g., since the beginning of lockdown during COVID-19, it is reported that there is an increase in symptoms such as difficulty in concentration (48%), memory loss (47%), and agitation/restlessness (45%) among the PLWD [123]. Furthermore, it is reported that symptoms of PLWD living alone have increased in comparison to those living with the carers since the beginning of lockdown during COVID-19, e.g., memory loss (54% vs. 42%) and difficulty in reading or writing (35% vs. 24%) [123]. In this systematic review, we have presented various types of devices to assist PLWD which can be used by carers in a remote setting, thereby reducing the health risks associated with physical contact while ensuring minimal impact on existing support mechanisms. Additionally, we overviewed the potential benefits and limitations of devices and associated services that can be used by PLWD to reduce infection risks during pandemics such as COVID-19. No articles on devices for PLWD specifically addressed the physical restrictions and other challenges imposed during COVID-19. Furthermore, we have identified the technological gaps in the existing assistive devices, which can be addressed in future research on remote care for PLWD.

In several studies examining the awareness of existing ATs, certain discrepancies were observed with regard to the awareness of available technologies for PLWD [30,31,32,121,125]. This lack of awareness of technology increases when the PLWD lives alone. Furthermore, these studies identify the gaps in awareness of new technological devices, irrespective of their usefulness to PLWD and their current commercial availabilities. Therefore, it is not clear whether recent technologies can be used to fulfil the needs of PLWD. This issue of awareness should be adequately addressed in future research, so that the technologies which offer clear benefits can reach PLWD and carers alike.

Several studies indicated a perception of stigma associated with AT devices for PLWD, and this may limit acceptance rates amongst potential users [33,36]. For example, acceptability of users for hip protectors is an issue, because of discomfort during use. The utility of an AT is highly dependent on the level of confidence it imparts on its users. If the users are apprehensive about the AT’s performance or have concerns about the requirement of personal help during its usage, then it is likely that PLWD will find the AT to be inconvenient. More research is required to ensure the devices adequately support specific scenarios created by pandemics, because when the acceptance rate of devices by the PLWD is low, then needs of PLWD and carers go unaddressed, in particular during lockdowns. Only when the users are aware of the technologies and accept them and find utility in daily lives, can remote access and support be applied on a wider scale, as required during the ongoing COVID-19 pandemic. Furthermore, if home visits by the carers become necessary, physical contact with the PLWD oftentimes cannot be avoided, which is an issue in a pandemic scenario such as COVID-19. Hence, more research is required in this area. There are certain devices that can aid the PLWD having language impairment, through social interactions and human computer interactions [100,103]; however, such devices need more systematic trials and user testing at this stage.

We have shown that AT can play a significant role in improving the lives of PLWD, but how the AT devices evoke different emotions among the users in order to provide a positive experience for the users is not heavily emphasized by the manufacturers of these devices [126]. Many companies producing AT devices do not engage in thorough testing of commercial products with the actual user. They focus mainly on the technological improvements and commercialization, whereas it is essential to discover and understand the needs of the users and the caregivers to improve and provide effective AT devices. Because of the complexity and diversity in the needs of the people with cognitive disabilities, it is highly essential that the AT devices are designed to support the changing needs of the users [127]. Hence, user-centred design and user testing should be an integral part of development of AT devices in order to improve the user experience. In order to increase the acceptance of devices, interviews with PLWD and carers may be crucial in determining user needs [125,128,129], where the user-centred design can be defined as an interactive design process in which the designers focus on the users and their needs in every phase of the design process. However, overreliance on a particular approach can be rather counterproductive to a clear understanding of user requirements. Focusing on subjective reporting alone may require the researcher to clarify and further validate findings, to reach a clear understanding of AT’s potential benefits. Furthermore, in order to develop an appropriate technology for PLWD, it is also essential to know the stages of dementia and levels of cognitive decline in the PLWD being assessed. At very severe levels of cognitive decline, PLWD may not accurately identify what they actually need. Moreover, designers also need to take into account cross-cultural studies, such as COGKNOW [130], in order to make sure that ATs are not culturally biased. It is not the usability that is to be targeted; rather, the devices should be resilient to the constraints imposed by the pandemic scenario, e.g., the devices should not rely on very high-speed internet connectivity in order to remotely monitor the activities of PLWD. The video monitoring devices which require cloud computing falls into this category of technological improvement [106].

More trials should be encouraged to investigate ways to reduce the COVID-19 virus transmissions in care homes, such as PROTECT, led by the University of Nottingham [29]. It has been reported that up to half of the deaths related to COVID-19 in the UK have occurred in care homes [29]. Moreover, recent attempts to certify the AT devices, such as the Certification-Dementia NW Europe project (CertD), should be encouraged among small- and medium-scale enterprises [131]. This Europe-wide programme focuses on thoroughly testing the usability of commercially available devices in living lab settings with PLWD. Importantly, the CertD will provide an EU certification to device SMEs which meet PLWD and caregiver requirements [131].

To summarize, this review presented existing AT for PLWD, and provided the pros and cons of the existing technical solutions for dementia patients that might be helpful during a pandemic which enforces restrictions on physical contact. Future directions in these areas of research which can improve the effectiveness of devices for PLWD and their carers have also been presented. User testing, emphasis on stages of dementia, and improving awareness about devices are important factors that require more effort in future research involving devices for dementia patients. User testing helps account for factors such as cultural biases and levels of cognitive decline during the design stage. In order to design and develop an appropriate technology for the PLWD and their carers, it is essential to take into account the stages of dementia and levels of cognitive decline of the PLWD being assessed. Moreover, higher awareness about these devices among PLWD and carers will help in improving the uptake of these devices. Manufacturers need to incorporate these factors as part of the design process. The main aspects of the several clinical trials conducted to study the utility of AT devices for dementia are also presented along with important requirements for future trials.

## Data Availability

Not appliable.
